# Simiao Pill Attenuates Collagen-Induced Arthritis in Rats through Suppressing the ATX-LPA and MAPK Signalling Pathways

**DOI:** 10.1155/2019/7498527

**Published:** 2019-03-14

**Authors:** Pan Shen, Shenghao Tu, Hui Wang, Kai Qin, Zhe Chen

**Affiliations:** Department of Integrated Chinese Traditional and Western Medicine, Tongji Hospital, Tongji Medical College of Huazhong University of Science and Technology, Wuhan, 430070, China

## Abstract

**Objective:**

Simiao pill (SM), a traditional Chinese formula, has been used as an antirheumatic drug in clinical practice for hundreds of years. Rheumatoid arthritis (RA) is characterized by chronic synovial inflammation and hyperplasia, cartilage destruction, and joint damage. This study was designed to investigate the protective effects of SM on collagen-induced arthritis (CIA) in rats. It also aimed to explore whether this protective effect of SM was related to the inhibition of the ATX-LPA and MAPK signalling pathways.

**Materials and Methods:**

Rats were injected with a collagen II emulsion at the end of the tail and on the back to induce arthritis. Treatment with different doses of SM was conducted by intragastric administration. Then, body weights and arthritis scores were measured. The serum levels of tumour necrosis factor (TNF)-*α*, interleukin (IL)-1*β*, C-reactive protein (CRP), osteoprotegerin (OPG), autotaxin (ATX), and lysophosphatidic acid (LPA) were determined by ELISA. Pathological changes in the joints were measured by micro-CT and assessed via haematoxylin-eosin (H&E) staining. The expression of ATX, LPA receptor 1 (LPA1) was detected by immunohistochemical staining, and the expression of mitogen-activated protein kinase (MAPK) was detected by Western blotting.

**Results:**

SM significantly alleviated arthritis symptoms, inhibited bone erosion, and decreased the levels of TNF-*α*, IL-1*β*, CRP, ATX, and LPA in the sera of CIA rats. Importantly, SM clearly reduced the protein expression of LPA1 and ATX. The activation of the MAPK signalling pathway was also inhibited by SM in the synovial tissues of CIA rats.

**Conclusions:**

The antirheumatic effects of SM were associated with the regulation of the ATX-LPA and MAPK pathways, the suppression of proinflammatory cytokine production, and the alleviation of cartilage and bone injury. These findings suggest that SM might be a promising alternative candidate for RA therapy.

## 1. Introduction

Rheumatoid arthritis (RA) is an autoimmune disease characterized by chronic systemic inflammation, synovial hyperplasia, cartilage destruction, and bone damage that leads to pain, swelling, and deformation of the joints [1]. Some patients present with pleuritis, arteritis, peripheral neuropathy, major depressive disorder, and other physical and mental diseases. RA usually causes disability or even death and is a considerable burden for the individual and society [2]. It is essential to develop novel treatment strategies to control inflammation and reduce consequent dysfunction.

The pathogenesis of RA is complicated; lymphocytes, plasma cells, and macrophages infiltrate the synovium form and cause synovial hyperplasia and the expression of proinflammatory cytokines such as IL-1*β*, IL-6, IL-17, and TNF-a, which have been reported to play a role in the pathogenesis of RA [3, 4]. The infiltration of these cytokines, immune cells, and fibroblast-like synoviocytes produces a pannus that leads to joint destruction. The suppression of proinflammatory mediators has been proposed as a therapeutic strategy against RA.

Lysophosphatidic acid (LPA) is the simplest glycerophospholipid and an important phospholipid signalling molecule that is involved in the regulation of diverse biological activities such as cell migration, proliferation, apoptosis, differentiation, and proinflammatory cytokine secretion [5]. Intracellular LPA is generated from cell membrane phosphatidic acid hydrolysis by the enzyme activities of phospholipase D (PLD) and phospholipase A2 (PLA2) [6]. LPA in bodily fluids is mainly generated by autotaxin (ATX) from circulating the lysophospholipid (LPC), and ATX participates widely in the pathological processes of various diseases [7, 8]. The ATX-LPA signalling pathway is a promising therapeutic target in diseases correlated with chronic inflammation including cancer and arthritis [9]. It has been reported that the genetic deletion of the ATX and LPA1 receptor [10] or the inhibition of ATX [11] relieves symptoms in an arthritis model. Moreover, LPA and ATX expression are significantly increased in the synovial tissues and synovial fluid of RA patients [12], suggesting that ATX-LPA signalling may be involved in RA.

Mitogen-activated protein kinases (MAPKs) are serine/threonine kinases that are known to regulate cellular apoptosis, differentiation, inflammation, and immunity [13, 14]. MAPK family members play a vital role in mediating continuous inflammation, synovial proliferation, pannus formation, and joint destruction in RA [15–17]. Therefore, the ATX-LPA and MAPK signalling pathways regulate inflammatory responses and are also regarded as suitable anti-inflammatory targets.

Currently, nonsteroidal anti-inflammatory drugs (NSAIDs), glucocorticosteroids, disease-modifying antirheumatic drugs (DMARDs), and biological agents that are widely approved for the alleviation of disease activity, synovial hyperplasia, and inflammatory cell infiltration and the reduction of bone destruction are commonly prescribed to treat RA. Unfortunately, NSAIDs cause various side effects such as gastrointestinal disorders and cardiovascular responses or can be toxic [18]. In addition, genetic and pathophysiological differences among RA patients limit the effects of these drugs. Thus, there is an urgent need to develop new potential therapeutic drugs for RA. Due to its therapeutic effects and fewer side effects, traditional Chinese medicine (TCM) has served as an important source of bioactive compounds that play a crucial role in the drug discovery and development processes. Medicines used in TCM can be used as complementary and alternative drugs for the treatment of RA [19].

Simiao pill (SM), a Chinese traditional formula composed of Cortex Phellodendri Chinensis (Huang Bai), Rhizome Atractylodis (Cang Zhu), Radix Achyranthis Bidentatae (Niu Xi), and Semen Coicis (Yi Yi Ren), eliminates heat and dampness and is therefore prescribed as an antirheumatic treatment in traditional Chinese medicine. Additionally, some studies have indicated that SM exerts many pharmacological effects such as anti-inflammation, detumescence, and pain relief  [20]. However, there have been no reports on the effect of SM in the development of RA and its relevant mechanisms. Therefore, the current study was designed to explore the effect of SM in collagen-induced arthritis (CIA) and to explore the role of ATX-LPA and MAPK signalling pathways in the progression of CIA.

## 2. Materials and Methods

### 2.1. CIA Animals

Fifty male Wistar rats weighing 150-190 g were provided by the Hubei Provincial Center for Disease Control and Prevention (Animal Certificate of Conformity: SCXK (Hubei) 2015-0018). The rats were kept under normal lighting conditions with six rats in each cage. Food and water were freely available, and the room temperature was kept at 22 ± 4°C. Rats were adaptively fed for one week before the experiment. Bovine type II collagen (CII) (Chondrex Inc. Redmond, WA, USA) was kept in a refrigerator at 4°C overnight. On the second day, collagen was mixed with complete Freund's adjuvant (CFA) (Sigma, USA) at a 1:1 v:v ratio in an ice-water bath to obtain a collagen emulsion and stored in a refrigerator at 4°C. Each rat was injected with 0.3 mL of the CII emulsion at the end of the tail and on the back, and a second injection was administered on the 7th day with an equal amount of emulsified CII. The rats in the control group were injected with an equal volume of saline, and the CIA model was evaluated [21]. The study programme was approved by the Ethical Committee of Tongji Hospital, Tongji Medical College, Huazhong University of Science and Technology (TJ-A20170502). The degree of arthritis was examined every 3 days. At the same time, the body weights of the rats were measured. Arthritis levels were graded using a scoring system as previously described, with a maximum score of 16 for every rat. The severity of the arthritis was expressed as a visual semiquantitative scoring system ranging from 0 to 4 per paw according to the following criteria: 0, normal joint; 1, slight swelling or erythema of one digit; 2, red skin and slight swelling of the ankle and foot; 3, modest swelling and erythema; and 4, severe swelling and erythema involving the entire hind paw or forepaw [22].

### 2.2. Simiao Pill Treatment

In this study, concentrated granules containing Cortex Phellodendri Chinensis, Rhizome Atractylodis, Radix Achyranthis Bidentatae, and Semen Coicis were obtained from Beijing Tcmages Pharmaceutical Co., Ltd. The herbs were extracted, and then the extracts were concentrated to form granules. This process was conducted according to Good Manufacturing Practice (GMP) for Drugs (Chinese FDA, 2010 Version) to guarantee herb quality. The efficacy of 1 g of Simiao granules was equal to that of 6 g of decoction pieces. Among the 50 rats injected with CII, 36 rats developed arthritis beginning at day 14. CIA rats were randomly divided into a CIA group, a methotrexate (MTX) group, a low-dose Simiao pill group (SM-L), a medium-dose Simiao pill group (SM-M), and a high-dose Simiao pill group (SM-H), with six rats in each group. MTX is one of the most commonly used DMARDs to treat rheumatoid arthritis and was used as a positive control in the present study. SM granules and MTX were dissolved in normal saline. Starting from the 14th day of primary immunization, rats in the SM group were given Simiao decoction at 8.63, 4.31, and 2.16 g/kg·d^−1^. The dose was derived from the human dose and converted to the equivalent rat dose based on body surface area. Rats in the SM group were given 15 ml/kg of SM decoction at 9 a.m. via intragastric administration once daily. The rats in the healthy control group (control) and the CIA group received the same volume of normal saline. For rats in the MTX group, MTX was administered orally once a week at 2 mg/kg [23]. The therapeutic effect of SM on arthritis was monitored by pathological changes of the joint tissues.

### 2.3. Tissue Collection

The rats were euthanized with an overdose of 2% pentobarbital and sacrificed on the 53rd day. Blood was taken from the abdominal aortas of the rats, serum was taken from the blood, and the serum was centrifuged at 3000 rpm for 10 minutes before being chilled at −80°C. Tissues from the knee joint synovium and ankles were removed. The ankles were placed in 4% paraformaldehyde for measurement.

### 2.4. Micro-CT Scanning

On the 53rd day of the experiment, the right ankle joints were surgically removed. The skin and muscle tissues were removed, and the bone tissues were fixed with 4% paraformaldehyde at room temperature. Three days later, the isolated ankle joints were scanned in a micro-CT machine. The samples were arranged and checked in a Sky Scan 1072 (SkyScan, Aartselaar, Belgium) scanner at an isometric resolution of 9 mm. SkyScan CT-analyser version 1.8 was used to make a three-dimensional reconstruction of the ankles, characterize the joint morphology, and inspect the effectiveness of SM on the CIA rats.

### 2.5. Ankle Tissue Pathology

The right rat hind limbs with their neighbouring tissues were fixed with 4% formaldehyde for 48 h and placed in a 10% EDTA solution for 45 days. A decalcification treatment was performed every two days. Decalcification was conducted for two months, and each group was then embedded in paraffin and cut into slices (4 *μ*m) before H&E staining. Pathological changes of the joints were detected with a light microscope (Olympus, Japan) and photographed. Pictures were taken at magnifications of 200× and 400×. Changes in inflammatory cell infiltration, synovial hyperplasia and lesions, and bone and cartilage damage in addition to other pathological changes were also detected. Histopathological changes were scored using the following criteria: 0: normal; 1: infiltration of inflammatory cells; 2: slight synovial hyperplasia; 3: moderate synovial hyperplasia or pannus formation, slight bone or cartilage damage; 4: severe synovial hyperplasia, moderate bone or cartilage damage; 5: severe inflammatory infiltration and joint destruction [24, 25].

### 2.6. ELISA for the Detection of Serum Levels of TNF-*α*, IL-1*β*, CRP, ATX, LPA, and OPG

Enzyme-linked immunosorbent assay (ELISA) kits to detect the expression levels of CRP (RA20041), osteoprotegerin (OPG) (RA20507), ATX (RA21333), and LPA (RA21341) were purchased from Bio-Swamp Co., Ltd. (Shanghai, China). ELISA kits for the detection of the serum proinflammatory cytokines TNF-*α* (ERC 102a) and IL-1*β* (ERC 007) were obtained from NeoBioscience Co., Ltd. (Shenzhen, China). The optical density at a wavelength of 450 nm in each well was recorded after five minutes using a multipurpose microplate reader (Synergy, BioTek, USA).

### 2.7. Immunohistochemistry Analysis

Paraffin sections were dewaxed and incubated for 10 minutes with a 3% H_2_O_2_ solution. Each section was incubated with goat serum at room temperature for 30 minutes and then incubated with primary antibodies against LPA receptor 1 (LPA1) (Abcam, USA) (1:100) and ATX (Abcam) (1:50) overnight at 4°C. Sections incubated in phosphate-buffered saline without antibody served as negative controls. Sections were incubated with a secondary antibody for 1 h and horseradish peroxidase for 30 minutes. The sections were then dyed with DAB reagent. A light microscope (Olympus, Japan) was used to magnify the ankle sections at 200× and 400× magnification. Image Pro Picture, image analysis software, was used for quantitative analysis. Sections were selected to measure positive cell absorbance.

### 2.8. Western Blotting

Synovial tissues were homogenized in RIPA buffer for 30 minutes and then centrifuged at 12000 rpm for 15 minutes at 4°C. The protein levels were determined using a bicinchoninic acid (BCA) assay kit (Good-bio, China). Protein samples (50 *μ*g/well) were separated by 12% polyacrylamide gel electrophoresis (SDS-PAGE), transferred to a PVDF membrane (Sigma, USA), and then blocked with 5% skim milk for 1 h. The membranes were incubated with antibodies against ERK1/2 (CST, USA) (1:1000), phosphorylated ERK1/2 (p-ERK1/2, CST) (1:1000), p38 (CST) (1:2000), phosphorylated p38 (p-p38, CST) (1:1000), JNK (CST) (1:1000), and phosphorylated JNK (p-JNK, CST) (1:1000) at 4°C overnight. *β*-Tubulin and GAPDH acted as internal controls. After washing three times with Tris-buffered saline containing Tween 20 (TBST), the PVDF membranes were incubated with a fluorescent secondary antibody 1 h at room temperature. Changes in the density of the protein bands were quantified with ImageJ software.

### 2.9. Statistical Analysis

The results are presented as the means ± SEMs. Data were analysed by one-way ANOVA. Comparisons between two groups were performed using Dunnett's multiple comparisons test. GraphPad Prism version 7.0 (GraphPad Software) was used for statistical analysis. P< 0.05 was considered statistically significant. P< 0.01 was considered more statistically significant.

## 3. Results

### 3.1. Effects of SM on Body Weight and Arthritis Score

**Figure 1:**
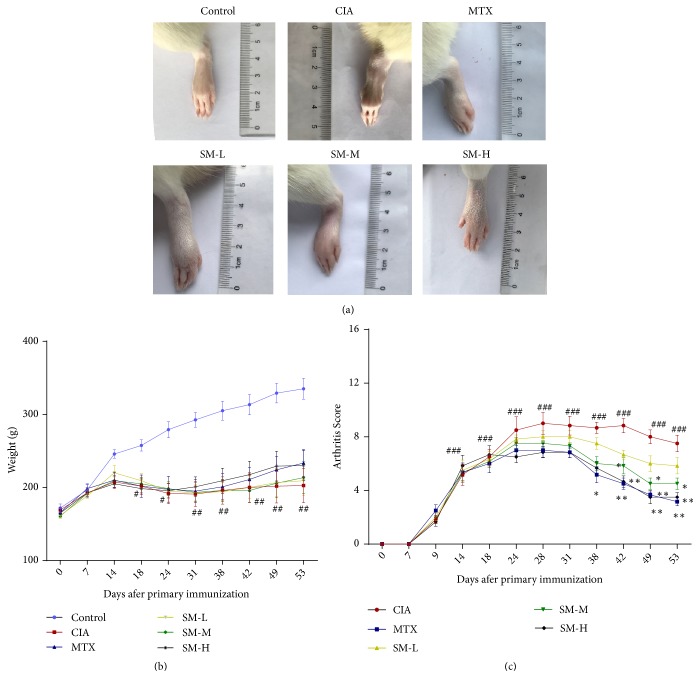
*SM attenuates the severity of arthritis in CIA rats*. (a) SM treatment suppressed paw swelling in rats with CIA. (b) The weights of rats in the SM treatment groups increased compared with those of rats in the untreated group, but this difference was not statistically significant. (c) SM significantly decreased the mean arthritis score in CIA rats compared to that in control rats. Data are expressed as the mean ± SEM (n=6). ^#^P< 0.05,^ ##^P< 0.01 compared with the control group; *∗*P< 0.05, *∗∗*P< 0.01 compared with the CIA group.

First, we investigated the effect of SM on the severity of arthritis in CIA rats. CIA rats were in poor conditions with sapless hair and exhibited lower food intake and dull responses compared to the control rats. Meanwhile, the weights of CIA rats decreased after the second immunization and then increased slowly (P< 0.05). However, differences in body weight between the high-dose SM group and the CIA group were not statistically significant (Figure 1(b)). The average arthritis score indicated that the collagen-induced arthritis model was successfully established (Figure 1(c)). On the 31st day, arthritis symptoms reached the first peak, and the difference between the CIA and control groups was significant (P< 0.01). Compared with that in the CIA group, the arthritis activity in the SM-treated group was alleviated beginning from the 38th day, and arthritis symptoms were remarkably decreased (P< 0.01), suggesting the antiarthritic effect of SM on CIA rats. As observed from physical state and behaviour, there were no significant adverse effects in the SM treatment groups.

### 3.2. Effect of SM against Joint Injury in CIA Rats

**Figure 2:**
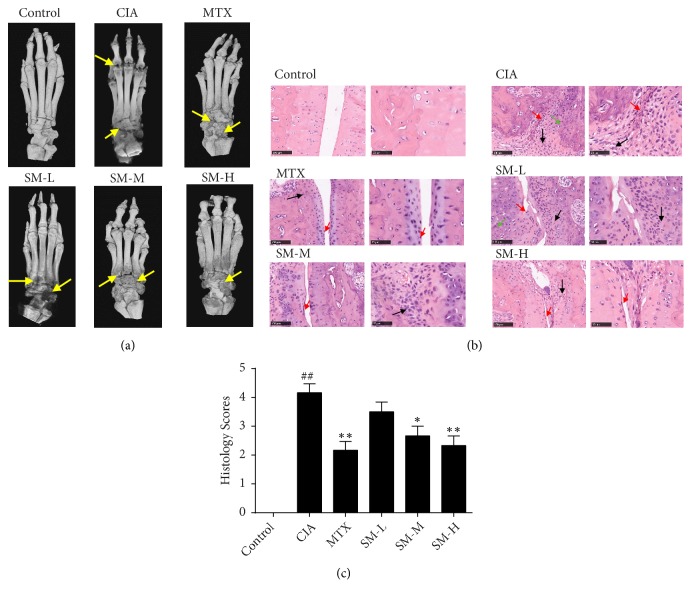
*SM alleviates joint inflammation and injury in CIA rats*. (a) Three-dimensional micro-CT images revealed that bone destruction (yellow arrows) was less robust in the SM-H group compared with that in the CIA group. Following SM treatment, changes in the ankle joints of rats from different groups, including interphalangeal joints, cavities, and bone, were assessed by micro-CT. (b) The histological assessment of ankle joints from different groups is shown. (c) The histology scores for synovial hyperplasia, pannus formation, and cartilage and bone damage were blindly evaluated. Values are the mean ± SEM (n = 6). ^#^P< 0.05,^ ##^P< 0.01 compared with the control group; *∗*P< 0.05, *∗∗*P< 0.01 compared with the CIA group.

Next, we analysed changes in the ankle joints and the surrounding tissues through the use of an in vitro micro-CT machine. The ankles of the CIA rats were seriously damaged, their interphalangeal joints were eroded, cavities were generated, and the bone was severely injured. Joint injuries in the high-dose SM-treated group and the MTX group were significantly improved compared with those in the CIA group (Figure 2(a)). Normal rat ankle histology showed complete synovial cells without synovial hyperplasia, inflammatory cell infiltration, or bone and cartilage damage (Figures 2(b)-2(c)). High-dose SM treatment significantly inhibited the typical pathological changes of ankle tissues seen in CIA rats (P< 0.01); this included a reduction in inflammatory infiltrating cells, the decrement of bone and cartilage damage, the alleviation of synovial cell propagation, and a decrease in the congestion and oedema of synovial tissues, as shown by histological analysis (Figure 2(b)).

### 3.3. Effect of SM on the Expression of the Proinflammatory Cytokines ATX, LPA, and OPG in Serum

**Figure 3:**
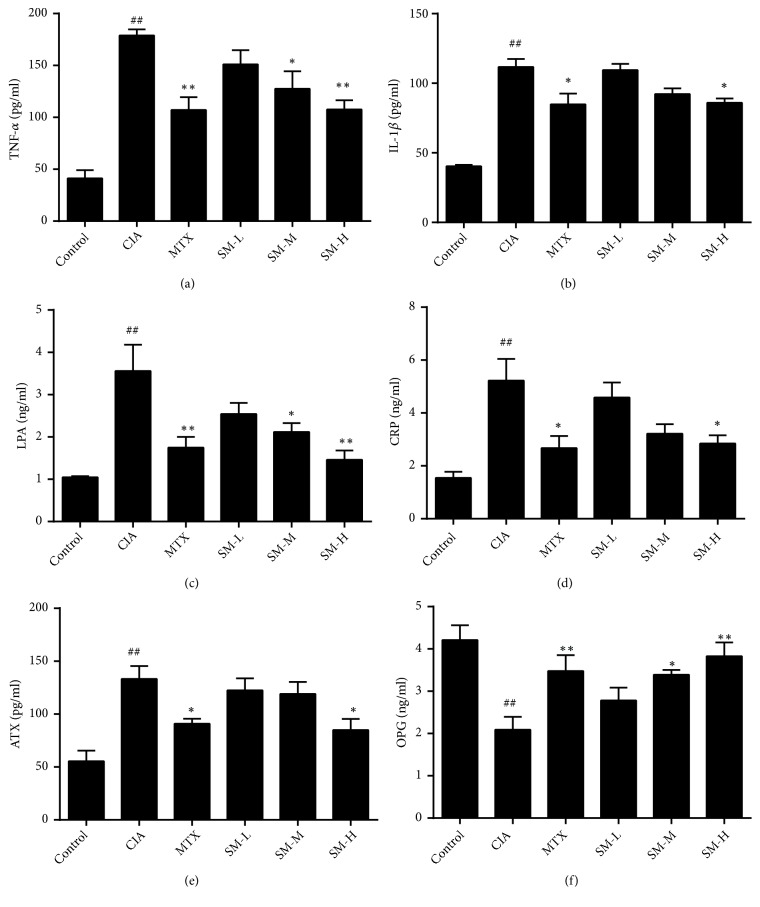
*SM decreases the expression of IL-1β, TNF-α, CRP, ATX, and LPA in the sera of CIA rats*. Serum levels of IL-1*β*, TNF-*α*, CRP, OPG, ATX, and LPA were measured by ELISA. All experiments were performed in triplicate. Values are the mean ± SEM (n = 6). ^#^P< 0.05, ^##^P< 0.01 compared with the control group. *∗*P< 0.05, *∗∗*P< 0.01 compared with the CIA group.

Proinflammatory cytokines play an important role in chronic inflammation, synovial hyperplasia, and bone damage in RA. Experiments indicated that the levels of TNF-*α*, IL-1*β*, CRP, OPG, ATX, and LPA in the sera of rats in the CIA group increased significantly (P< 0.01) compared to those in rats in the control group (Figures 3(a)–3(e)), whereas, as shown in Figure 3(f), the OPG content decreases significantly (P< 0.01). The serum concentrations of TNF-*α*, IL-1*β*, CRP, ATX, and LPA in the high-dose SM group and the MTX group were significantly decreased compared with those in the CIA group (P< 0.01, P< 0.05), while the level of OPG in the SM groups (high-dose and medium-dose) increased significantly (P< 0.01, P< 0.05). These findings suggested that SM inhibited cytokine production, reduced joint synovitis, and prevented synovial cells from excreting proteases to reduce joint damage.

### 3.4. Effect of SM on the Expression of ATX and LPA1 in Ankle Tissues

**Figure 4:**
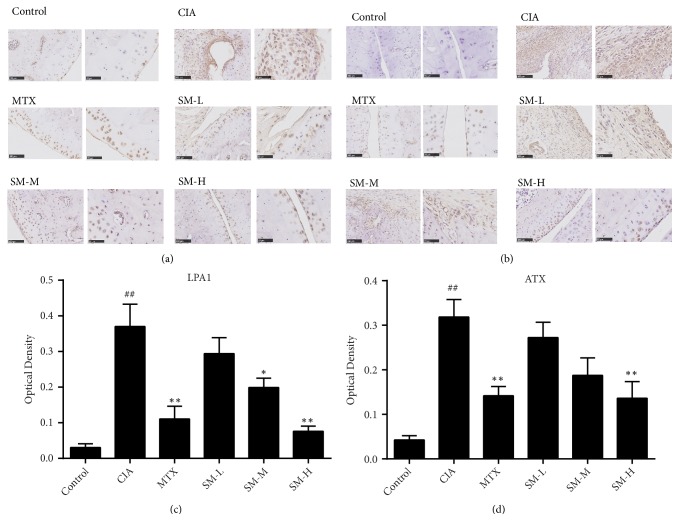
*SM reduces the expression of LPA1 and ATX in ankle tissues*. Immunohistochemical staining acquired from ankle joints of the several groups (a) with LPA1 detection and (b) ATX detection. OD measurements of LPA1 (c) and ATX (d) were analysed. Values are the mean ± SEM (n = 6). ^#^P< 0.05, ^##^P< 0.01 compared with the control group. *∗*P< 0.05, *∗∗*P< 0.01 compared with the CIA group.

As shown in Figures 4(a) and 4(c), compared with that in rats in the control group, the expression of LPA1 in the ankle tissues was significantly increased in rats in the CIA group (P< 0.01). LPA1 expression was the lowest in the SM-H group (P< 0.01). Compared to the optical density (OD) of the CIA group, the OD of the MTX group was remarkably declined, but the OD of the MTX group was higher than that in the SM-H group. As shown in Figures 4(b) and 4(d), the expression of ATX was significantly higher in the CIA group than in the control group (P< 0.01). ATX expression was reduced in rats in the MTX and SM-H groups compared to that in rats in the CIA group (P< 0.05).

### 3.5. Effect of SM on the Activation of MAPK Signalling in Synovial Tissues

**Figure 5:**
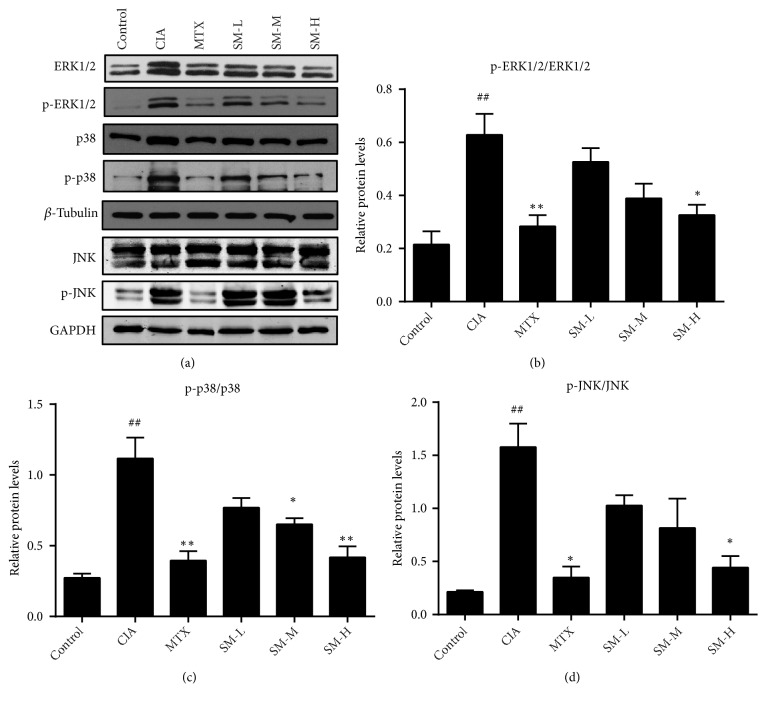
*SM suppresses the activation of MAPK signalling in the synovial tissues of CIA rats*. (a) SM suppressed p-ERK, p-p38, and p-JNK activation in the synovium of CIA rats, as shown by Western blot. The expression of (b) p-ERK, (c) p-p38, and (d) p-JNK in the different groups was assessed by semiquantitative analysis. Values are the mean ± SEM (n = 3). ^#^P < 0.05, ^##^P < 0.01 compared with the control group. *∗*P< 0.05, *∗∗*P< 0.01 compared with the CIA group.

Finally, we explored the molecular mechanisms involved in the antiarthritic effects of SM on CIA rats. As shown in Figure 5, the expression levels of p-ERK1/2, p-p38, and p-JNK in the synovial tissues of rats in the CIA group were significantly higher than those in rats in the control group (P< 0.01). Following high-dose SM treatment, the phosphorylation of ERK1/2, p38, and JNK was obviously downregulated compared to that in rats in the MTX group (P< 0.05), suggesting the involvement of MAPK signalling inhibition in the antiarthritic effects of SM on CIA rats.

## 4. Discussion

RA is a chronic autoimmune disease involving pain, stiffness and inflammation of the joints, and cartilage and bone damage that ultimately leads to functional disability. The aetiology and pathological changes of RA are not completely clear, so effective and safe treatment methods to prevent and cure RA are still lacking. Current treatments are aimed at the inflammatory cytokines and signalling pathways in the pathogenesis of RA. Due to the complexity of the inflammatory network, even if patients use therapeutic drugs against a single type of cells, inflammatory factors, or signalling pathways, the clinical effect is still insufficient. Side effects are also problematic and cannot be ignored. The therapeutic goal is to find effective and safe agents that can reduce joint damage and complications and delay the progression of RA.

SM is a TCM formula that removes heat and dampness that has been used to treat arthritis in China since the Qing Dynasty. SM can regulate blood lipids and was originally used for the treatment of metabolic abnormal arthritis, and it has been formulated over nearly 400 years of clinical practice to minimize the systemic side effects. Previous studies showed that Simiao pill could exert potential anti-inflammatory effects in vitro [26] and inhibit glomerular inflammation and proteinuria in a rat model [27]. However, the effects of SM in the treatment of CIA are rarely reported. In the present study, we successfully established a CIA model in rats that shared many pathological characteristics with RA, including swelling, pain, weight loss, inflammation, proliferation of the synovium, and damage to bone and cartilage. We investigated the beneficial effect of SM on joint histology and joint function in CIA rats. SM significantly improved arthritis symptoms; increased the level of OPG; inhibited the levels of TNF-*α*, IL-1*β*, CRP, ATX, and LPA; and alleviated cartilage and bone injury.

The dysregulation of cytokines including TNF-*α* and IL-1*β* contributes to inflammation and is responsible for synovial hyperplasia and progressive joint destruction [28]. In RA patients, TNF-*α* and IL-1*β* play a vital role at each stage of RA pathogenesis by strengthening immune responses, provoking the release of other inflammatory cytokines, stimulating the differentiation of osteoclasts, and promoting joint damage, which begins a vicious cycle [29, 30]. Therefore, in the present study, we focused on changes in immune factors in CIA rats. The expression of TNF-*α* and IL-1*β* was increased in the serum of CIA rats compared to that in the serum of control rats. This increase in expression was inhibited by SM treatment, suggesting a role for SM in controlling chronic inflammation.

Increasing numbers of studies have demonstrated that the activation of the MAPK pathway serves as a risk factor for the persistence and progression of RA and is related to inflammatory responses to induce the expression of various inflammatory genes [31, 32]. The activation of proinflammatory cytokines including TNF-*α* and IL-1*β* depends on the upregulation of MAPKs to induce a significant chronic, inflammatory response [33]. We found that the activation of MAPKs was inhibited by SM in the synovial and spleen tissues of CIA rats.

In recent years, the important effect of the ATX-LPA pathway in the development of RA has been recognized. There are two main pathways that participate in LPA production. PLD and PLA2 are mainly involved in cellular LPA production from membrane phospholipids. Intracellular LPA is regarded as an intermediate in phospholipid synthesis; it is not likely that it acts as an extracellular pool of signalling molecules [6]. Extracellular LPA in biological fluids is mainly produced from LPC. LPC is generated from membrane phosphatidylcholine (PC) by the enzymatic action of PLA2. LPC is subsequently converted to LPA by the enzymatic action of ATX, a plasma lysophospholipase D (lysoPLD), which is considered the main source of extracellular LPA production [34]. It has been shown that the LPA levels in heterozygous ATX knockout mice were reduced by 50% compared to those in wild-type mice [35, 36]. In RA, ATX could induce the local production of LPA from LPC in the synovium and synovial fluid.

LPA levels rapidly decreased after the oral administration of an ATX inhibitor, which indicates that LPA is rapidly generated and degraded in vivo [37]. LPA1-6 can associate with a G protein-coupled receptor, leading to the inhibition of the phosphatidylinositol 3-kinase/Akt and Ras/MAPK pathways and the activation of RhoA GTPase. Through its control of these pathways, LPA can regulate various cellular processes including proliferation, differentiation, and apoptosis [38]. The ATX-LPA axis mediates many disorders, such as cardiovascular disease, cancer, and RA. Increasing evidence indicates that the activation of the ATX-LPA axis contributes to the progression of RA. ATX and LPA levels are elevated in the synovium and synovial fluid of patients with RA compared to those in healthy subjects [12], and LPA1-6 were detected in fibroblast-like synoviocytes (FLSs) in patients with RA. LPA1 was the main receptor expressed [39]. Joint swelling, synovial inflammation, cartilage damage, and bone erosion in LPA1-deficient CIA mice were significantly alleviated [11, 40]. LPA binds to receptors on FLSs in RA, stimulates the activation of the MAPK and Rho signalling pathways, regulates the production of cytokines, and rearranges the actin cytoskeleton in FLSs [11]. During the pathogenesis of RA, inflammation stimulates local, TNF-driven ATX expression in the synovium, inducing the hydrolysis of LPC and the production of LPA [41]. In turn, LPA activates FLSs and amplifies pathogenetic responses in synergy with TNF, which may induce further ATX expression and increase LPA production [42]. LPA indirectly stimulates osteoclasts through increasing cytokine levels, and the increase in cytokine by LPA facilitates osteoclast differentiation and synovial hyperplasia [43]. RA patients also showed increases in ATX and cytokine production in synovial fluid and in FLSs treated with LPA [40]. Overall, blocking the ATX-LPA pathway could produce certain therapeutic benefits, such as the reduction of FLSs proliferation and inflammatory cytokine production and a reduction in synovial hyperplasia, which are key elements that could reverse deformities and disability in RA. The ATX-LPA pathway represents a novel therapeutic target for the treatment of RA. Therefore, the inhibition of the ATX-LPA pathway is a promising strategy for preventing joint inflammation and destruction. The discovery of the effect of SM in a novel pathway of the ATX-LPA axis could help determine a strategy for CIA treatment. In the current study, the increase of ATX-LPA axis activity in CIA was attenuated by SM. Downregulated ATX and LPA1 levels in the synovial tissue compared to those observed without SM treatment might be associated with the inhibition of inflammatory cytokines by SM. The effects of LPA on FLSs activation and hyperplasia are known to be dependent on MAPK signalling [40]. SM may inhibit MAPK expression by regulating LPA. The current results might explain the protective effect of SM treatment on synovial inflammation and bone destruction in CIA rats, and the ATX-LPA and MAPK signalling pathways play an important role in these effects.

As an important enzyme for the formation of LPA, PLA2 also has a certain effect on the pathogenesis of RA [44]. Enhanced PLA2 activity was detected in the synovial fluid, peripheral blood polymorphonuclear leukocytes, and monocytes of RA patients [45]. Treatment of hTNF^+/-^ arthritis [46] and CIA [47] with an inhibitor of PLA2 attenuated arthritic symptoms and the inflammatory reaction. These studies demonstrated the positive role of active phospholipids and their related enzymes in the development of RA. Next, we will focus further research on components of the LPA production pathways, including PLA2 and ATX.

## 5. Conclusions

In summary, SM exhibited anti-inflammatory and joint protective effects in CIA rats. No obvious systemic adverse reactions were observed following SM treatment. These findings suggest that SM is a promising agent to reduce arthritis and bone loss by inhibiting the ATX-LPA and MAPK signalling pathways. Perhaps we can delay the progression of RA by regulating blood lipids and including TCM treatment. However, the underlying mechanisms of these results are still largely unexplored, and further studies are needed to clarify the details. We also need to determine and confirm all components of Simiao pill in addition to their metabolism and adverse effects in the treatment of RA.

## Data Availability

The data used to support the findings of this study are available from the corresponding author upon request.

## Abbreviations

RA: Rheumatoid arthritis
CIA: Collagen-induced arthritis
SM: Simiao pill
LPA: Lysophosphatidic acid
ATX: Autotaxin
PLA2: Phospholipase A2
MAPK: Mitogen-activated protein kinase
H&E: Haematoxylin-eosin
ERK: Extracellular signal-regulated kinase
JNK: c-Jun N-terminal kinase
CRP: C-reactive protein
MTX: Methotrexate
SM-L: Low-dose Simiao pill group
SM-M: Medium-dose Simiao pill group
SM-H: High-dose Simiao pill group
FLSs: Fibroblast-like synoviocytes.
